# CDs/FeCo-ONSs Composite with Peroxidase-like Activity for Ascorbic Acid Detection

**DOI:** 10.3390/nano16100634

**Published:** 2026-05-20

**Authors:** Xue Liu, Yuanhang Wei, Wenjing Wang

**Affiliations:** College of Chemistry and Chemical Engineering, Qingdao University, Qingdao 266071, China; liuxue2@qdu.edu.cn (X.L.); weiyuanhang@qdu.edu.cn (Y.W.)

**Keywords:** carbon dots, iron-cobalt oxide nanosheets, nanozyme, ascorbic acid detection

## Abstract

Nitrogen-doped carbon dots (CDs) were fabricated via a one-pot hydrothermal route using hydroquinone and o-phenylenediamine as dual precursors. The as-prepared CDs were then anchored onto iron-cobalt oxide nanosheets (FeCo-ONSs) to construct a composite nanozyme, denoted as CDs/FeCo-ONSs. Although FeCo-ONSs possess intrinsic peroxidase-like (POD-like) activity, the integration of CDs with FeCo-ONSs resulted in a remarkable enhancement of catalytic performance. Specifically, in the presence of hydrogen peroxide (H_2_O_2_), the CDs/FeCo-ONS composite promoted the efficient oxidative transformation of 3,3′,5,5′-tetramethylbenzidine (TMB), leading to the formation of a blue-colored oxidized product. Based upon the enhanced POD-like activity of CDs/FeCo-ONSs, a highly sensitive colorimetric sensor was developed for the detection of ascorbic acid (AA). This method exhibited a wide linear detection range of 0.1 to 50 µM with a low limit of detection (LOD) of 0.018 µM. Furthermore, the developed method was successfully applied to the determination of AA in commercial beverages and fresh fruits, verifying its potential feasibility for practical applications in food quality control.

## 1. Introduction

Nanozymes are an emerging class of functional materials that integrate the characteristics of nanomaterials with enzyme-mimetic catalytic activity. Compared with natural enzymes, nanozymes possess several distinct advantages, including high stability, facile recoverability, and low-cost synthesis [1,2]. Among various types of nanozymes, peroxidase mimics constitute one of the most extensively studied and applied subgroups, capable of efficiently catalyzing peroxidase-type reactions [3]. These robust artificial enzymes have been widely employed in biosensing, medical diagnostics, and therapeutic [4,5,6].

Carbon dots (CDs) are novel fluorescent carbon-based nanomaterials [7], featuring excellent water solubility, biocompatibility, chemical stability, and photoluminescence properties. These properties have enabled their broad application in cellular imaging, anti-counterfeiting, drug delivery, and sensing [8,9,10,11]. Heteroatom doping can effectively modulate the physicochemical properties of carbon dots [12]. Among various heteroatoms, nitrogen is the most widely adopted doping element for CDs, and nitrogen doping acts as a highly effective strategy to tailor the physicochemical properties of CDs. This is primarily attributed to its atomic size matching that of carbon and its capability to form diverse nitrogen-containing functional groups on the CDs. Such structural modulation not only remarkably enhances the electronic and optical properties of CDs [13,14], but also broadens their application potential in biomedicine and environmental detection [15,16]. Guo et al. synthesized blue-emitting nitrogen-doped carbon dots for the detection of intracellular Fe^3+^ [17]. Jiang et al. fabricated highly fluorescent nitrogen-doped carbon dots via a one-step sintering process and utilized the fluorescence quenching induced by p-nitrophenol to construct a sensitive glyphosate detection platform [18].

Owing to the high specific surface area, abundant active sites, and remarkable catalytic performance [19,20,21,22], two-dimensional (2D) nanomaterials have garnered widespread application in sensing, biomedicine, and other fields [23,24,25,26]. As a typical 2D nanomaterial, FeCo-ONSs possess a 2D sheet-like structure that maximizes the exposure of surface-active sites and demonstrates intrinsic peroxidase-like activity [27], making them suitable for constructing colorimetric and electrochemical biosensors [28]. Su et al. developed a sensitive dual-channel sensing system based on FeCo-ONSs and gold nanoclusters, enabling sensitive detection of melatonin through both fluorescence and colorimetric readouts [29].

Ascorbic acid (AA) is an essential water-soluble micronutrient that plays a pivotal role in biological processes, including antioxidant defense, collagen biosynthesis, and immune regulation [30,31]. As a common food nutrient fortifier and a pharmaceutical active ingredient, its quantitative determination represents a crucial parameter for evaluating food nutritional quality, clinical diagnosis, and pharmaceutical quality [32,33]. These applications necessitate the development of AA detection methods with high sensitivity, high selectivity, rapid response, and operational simplicity. A variety of methods have been established for AA detection, such as electrochemical methods [34] and chromatographic methods [35], but these require sophisticated instrumentation and specialized expertise. Over the past few years, colorimetric methods have gained increasing attention due to their simplicity, rapid response, and high sensitivity [36,37,38], positioning them as one of the most promising approaches for AA detection with broad application.

Although nanozyme-based colorimetric sensors have broad application prospects, there are still two major challenges that urgently need to be solved: low catalytic activity caused by poor electron transfer efficiency, and reduced exposure of active sites due to the agglomeration of metal-based nanosheets. To address these issues, we herein report an interface regulation strategy by combining yellow-emitting CDs with FeCo-ONSs. Specifically, CDs were synthesized using *o*-phenylenediamine and hydroquinone as precursors and then composited with FeCo-ONSs via an ultrasonic-assisted impregnation method. The obtained CDs/FeCo-ONSs nanocomposites exhibited significantly enhanced peroxidase-like activity compared with pure FeCo-ONSs or individual CDs. The key innovation of our method lies in the dual role of CDs: they can act as electron transfer mediators, where the high electrical conductivity of CDs promotes charge transfer between the active sites of FeCo-ONSs and reaction substrates, reducing the activation energy, and they can also serve as anti-agglomeration agents, where CDs inhibit the stacking of FeCo-ONSs during the catalytic process and retain more accessible active sites. This interfacial synergistic effect is essentially different from the previously reported physical mixture or simple co-incubation methods. Benefiting from this design, we constructed a highly sensitive and specific AA detection platform with a detection limit as low as 0.018 μM. We believe that this interface regulation strategy opens up a general approach for the development of high-performance nanozymes and has great potential in the field of food quality monitoring.

## 2. Materials and Methods

### 2.1. Materials and Apparatus

Hydroquinone, *o*-phenylenediamine (98.0%), iron(III) nitrate nonahydrate (Fe(NO_3_)_3_·9H_2_O), cobalt(II) chloride hexahydrate (CoCl_2_·6H_2_O), cetyl trimethylammonium bromide (CTAB), sodium borohydride (NaBH_4_), sodium hydroxide (NaOH), H_2_O_2_ solution (H_2_O_2_, 30% (*w*/*w*) in H_2_O), sodium acetate (NaAc, ≥99.0%), acetic acid (HAc), and ascorbic acid (AA) were purchased from Sinopharm Chemical Reagent Co., Ltd. (Shanghai, China). The 3,3′,5,5′-tetramethylbenzidine (TMB, ≥99.0%) was obtained from Aladdin (Shanghai, China). Terephthalic acid (TA, 99.0%) and rhodamine B (Rh B) were supplied by Macklin (Shanghai, China). All the above reagents are of analytical grade.

High-resolution transmission electron microscopy (HRTEM) measurements were performed using a JEOL JEM-2100F (JEOL, Tokyo, Japan) at an accelerating voltage of 200 kV. The elemental composition was analyzed by X-ray photoelectron spectroscopy (XPS; D8 Advance, Bruker, Germany) with Al Ka radiation (hν = 1486.6 eV). X-ray diffraction (XRD) patterns were obtained at ambient temperature (25 ± 2 °C) on a Smart Lab 3 KW diffractometer (Rigaku Corporation, Tokyo, Japan) using Cu Kα radiation (λ = 1.5406 Å). The scanning range was set from 5° to 80° with a scanning rate of 5°/min. The surface structure was analyzed using a Fourier transform infrared (FT-IR) spectrometer (Thermo Scientific, Nicolet Is50, Waltham, MA, USA) over the spectral range of 4000–400 cm^−1^ at a resolution of 4 cm^−1^. Ultraviolet-visible (UV-Vis) spectra were recorded with a UV-2700 spectrophotometer (Rigaku Corporation, Tokyo, Japan) in the wavelength range of 200–800 nm at a scan speed of 200 nm/min. Fluorescence measurements were conducted using a fluorescence spectrophotometer (F-7000, Hitachi Ltd., Tokyo, Japan). Both excitation and emission slit widths were set to 10 nm, and the photomultiplier tube voltage was 500 V. Atomic force microscopy (AFM) measurements were performed using a Bruker Dimension Icon atomic force microscope (Bruker, Karlsruhe, Germany) in tapping mode at ambient temperature. The scan area was set to 5 × 5 μm^2^ with a scan rate of 1.0 Hz. Nitrogen adsorption–desorption isotherms were measured using a specific surface area and porosity analyzer (ASAP 2460, Micromeritics, Norcross, GA, USA) at liquid nitrogen temperature (77 K). The Brunauer–Emmett–Teller (BET) method was applied to calculate the specific surface area. Time-resolved photoluminescence (TRPL) decay spectra were recorded using a fluorescence lifetime spectrometer (Edinburgh Instruments FLS1000, Livingston, UK) with a pulsed laser diode (λ = 375 nm, pulse width ≈ 100 ps) as the excitation source. The emission was monitored at the peak wavelength, and the decay curves were analyzed by deconvolution with the instrument response function (IRF).

### 2.2. Synthesis of CDs

Firstly, 30 mg of *o*-phenylenediamine and 30 mg of hydroquinone were dissolved in 10 mL of deionized water with magnetic stirring. The homogeneous solution was then transferred into a polytetrafluoroethylene reaction kettle and incubated at 180 °C for 12 h in a constant-temperature oven. After naturally cooling down to ambient temperature, the resulting dark brown solution was subjected to centrifugation at 8000 rpm for 8 min to remove aggregated large particles. Then, the supernatant was further purified through a 0.22 μm filter membrane to remove residual impurities. Further purification was achieved through dialysis (MWCO: 100–500 Da) against deionized water for 12 h. The resulting product was subjected to freeze-drying to afford a dry powder for subsequent use.

### 2.3. Preparation of FeCo-ONSs

A mixture of 0.202 g of Fe(NO_3_)_3_·9H_2_O, 0.119 g of CoCl_2_·6H_2_O, and 0.250 g of CTAB was dissolved in 12 mL of deionized water. After stirring continuously at ambient temperature for a duration of 30 min, freshly prepared NaBH_4_ (0.015 g/mL, 5 mL) aqueous solution was slowly added dropwise, followed by sustained stirring of the mixture for an additional 10 min. Subsequently, the crude product containing FeCo-ONSs was then collected via centrifugation at 8000 rpm for 5 min, and the obtained precipitate was thoroughly washed three times using ethanol. Finally, the obtained precipitate was dried at 60 °C under vacuum in a drying oven for 12 h to form a solid sample for subsequent use.

### 2.4. Preparation of CDs/FeCo-ONSs

Next, 60 mg of CDs were dispersed in 15 mL of absolute ethanol under magnetic stirring, and a transparent brown solution was formed. Subsequently, 30 mg of FeCo-ONSs were added to the solution, followed by ultrasonic treatment for 1 h. The resulting crude product was rinsed with ethanol six times, subjected to centrifugation, and then redispersed. Finally, the purified product was dried at 60 °C under vacuum in a drying oven for 12 h to form a solid powder.

### 2.5. Exploration of the POD-like Activity of CDs/FeCo-ONSs

To explore the POD-like activity of CDs/FeCo-ONSs, a colorimetric assay was carried out based on the catalytic oxidation reaction of TMB. Typically, 200 μL of 5 mM TMB was added to the reaction system comprising 500 μL of CDs/FeCo-ONSs solution (10 μg/mL), 300 μL of NaAc-HAc buffer (pH = 3.5), and 500 μL of H_2_O_2_ solution (50 μM). The reaction was then incubated in a thermostatted water bath at 50 °C for 35 min, and the absorbance intensity (λ = 652 nm) was measured. The influences of critical experimental parameters on the POD-like activity of CDs/FeCo-ONSs, including pH (3.0, 3.5, 4.0, 4.5, 5.0, and 5.5), reaction time (10, 15, 20, 25, 30, 35, and 40 min), and reaction temperature (25, 30, 35, 40, 45, 50, and 55 °C), were systematically investigated. All experimental measurements were performed in triplicate, and the average values were reported. The relative activity among different samples was calculated according to the following formula:

$$relative\;activity\left(\%\right)=\frac{A-A_{0}}{A_{max}-A_{0}}\times 100\%,$$

where *A* is the absorbance recorded for each sample, *A*_0_ is the absorbance of the blank sample (without H_2_O_2_), and *A_max_* is the maximum absorbance obtained among all tested samples.

### 2.6. Detection of Hydroxyl Radicals (·OH) by TA

To explore the POD-like catalytic mechanism between CDs/FeCo-ONSs and H_2_O_2_, fluorescence measurements were carried out to detect the generation of ·OH in the catalytic process. TA served as a specific capture probe for ·OH, forming highly fluorescent 2-hydroxyterephthalic acid. CDs (10 μg/mL), FeCo-ONSs (10 μg/mL), and CDs/FeCo-ONSs (10 μg/mL) were individually added to a solution containing NaAc-HAc buffer (pH = 3.5), TA (10 mM), and H_2_O_2_ (50 mM) for co-incubation. After 1 h of incubation in the dark at ambient temperature, the mixtures were centrifuged, and then the supernatant was subjected to fluorescence measurement, with the excitation/emission wavelength set at 315/426 nm.

### 2.7. Oxidation Experiment of Rhodamine B (RhB)

To further confirm that the POD-like activity of CDs/FeCo-ONSs arises from the catalytic H_2_O_2_ decomposition into ·OH, RhB was employed as a probe to detect the formation of ·OH. RhB (25 μM) was mixed with CDs/FeCo-ONSs at varying concentrations (10, 20, 30, 40, and 50 μg/mL). The reaction was then initiated by adding H_2_O_2_ (50 mM). After incubation of the mixture in the dark at ambient temperature for 6 h, the absorbance intensity at λ = 554 nm was measured.

### 2.8. Steady-State Reaction Kinetics Experiments of CDs/FeCo-ONSs

Steady-state reaction kinetics experiments of CD/FeCo-ONS nanocomposites were performed to assess the POD-like catalytic activity of CDs/FeCo-ONSs by measuring their oxidation of the chromogenic substrate TMB with H_2_O_2_. For TMB kinetics measurements, the experiments were performed in NaAc-HAc buffer solution at pH 3.5, in the presence of 50 μM of H_2_O_2_ and 10 μg/mL of CDs/FeCo-ONSs, while the TMB concentration was adjusted in the range of 1–5 mM. For H_2_O_2_ kinetics measurements, utilizing H_2_O_2_ as the substrate, all conditions were maintained the same as described above except that 5 mM of TMB was employed and the concentration of hydrogen peroxide (0.2, 0.25, 0.3, 0.5, 1, and 10 μM) was adjusted. The absorbance intensity at a wavelength of 652 nm was measured. The Michaelis–Menten equation was applied to estimate the Michaelis constant (K_m_) and maximum reaction rate (*V_max_*) of CDs/FeCo-ONSs:

$$V_{0}=\frac{V_{max}\cdot \left[S\right]}{K_{m}+\left[S\right]},$$

where [*S*] represents the substrate concentration of TMB or H_2_O_2_, and *V*_0_ is the initial reaction rate.

The double reciprocal form of the Michaelis–Menten equation is as follows:

$$\frac{1}{V_{0}}=\frac{K_{m}}{V_{max}}\frac{1}{\left[S\right]}+\frac{1}{V_{max}},$$

where *V_max_* and *K_m_* can be calculated from the intercept and the slope of the double reciprocal plot (also known as the Lineweaver–Burk plot), respectively.

### 2.9. Detection of H_2_O_2_ and AA

For the colorimetric detection of H_2_O_2_, TMB (5 mM), NaAc-HAc buffer solution (pH = 3.5), CDs/FeCo-ONSs (10 μg/mL), and different concentrations (0 to 50 μM) of H_2_O_2_ solutions were added into a centrifuge tube. After incubation at 50 °C in a water bath for 35 min, the absorbance intensity at a wavelength of 652 nm was measured.

For the colorimetric detection of AA, TMB (5 mM), NaAc-HAc buffer solution (pH = 3.5), CDs/FeCo-ONSs (10 μg/mL), and H_2_O_2_ (50 μM) were first added to a centrifuge tube and incubated in a 50 °C water bath for 35 min. Subsequently, a series of AA solutions with the concentration of 0.1–50 μM were added to the reaction system, followed by incubation at ambient temperature for 10 min to achieve the complete reaction of AA with CDs/FeCo-ONSs. The absorbance at a wavelength of 652 nm of the reaction system was accurately measured. The limit of detection (LOD) and limit of quantification (LOQ) for H_2_O_2_ and AA were calculated using the following formulas:

$$LOD=\frac{3\sigma }{s},$$

$$LOQ=\frac{10\sigma }{s},$$

where *σ* is the standard deviation of ten blank measurements, and *s* is the slope of the calibration curve.

### 2.10. Detection of AA in Beverages and Fruits

To verify the practical applicability of the CD/FeCo-ONS-based colorimetric assay for determining AA in real samples, three commercial beverages (Nongfu Spring C100 Juice, Master Kong Daily C Peach Juice, and Minute Maid Orange Juice) and three fresh fruits (litchi, kiwi, and orange) were selected as test samples. In this study, considering the intrinsic AA content of commercial beverages and fresh fruits, all samples were first diluted 40- to 200-fold to bring the endogenous AA concentration into the linear detection range. Subsequent spike-and-recovery experiments were performed to further eliminate potential systematic errors introduced by high dilution factors and verify the accuracy of the sensor response toward AA in diluted matrices. The selected spiking concentrations were carefully chosen to ensure that the final total concentration of the mixed solutions remained within the optimal linear interval of the calibration curve.

For beverage samples, each product was first centrifuged, filtered, and appropriately diluted. For fruit samples, the edible portions (150 g) were homogenized using a grinder. The resulting homogenate was centrifuged at 8000 rpm for 8 min, after which the supernatant was passed through a 0.22 μm filter membrane and diluted with deionized water.

The colorimetric detection was carried out by first incubating a mixture of TMB (5 mM), NaAc-HAc buffer solution (pH = 3.5), CDs/FeCo-ONSs (10 μg/mL), and H_2_O_2_ (50 μM) at 50 °C for 35 min in a water bath. After that, either standard AA solutions with different concentrations or 500 μL of beverage/fruit extract samples were introduced into the system. After incubation at ambient temperature for 10 min, the absorbance intensity at a wavelength of 652 nm was measured.

## 3. Results and Discussion

### 3.1. Characterization

**Figure 1:**
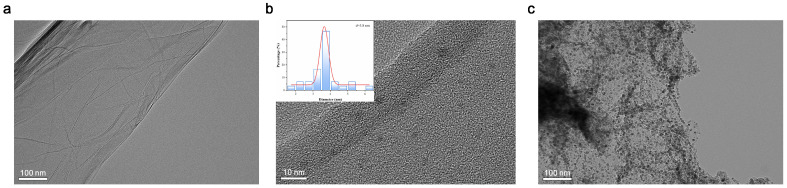
HRTEM images of the synthesized FeCo-ONSs (**a**), CDs (insert: corresponding particle size distribution histogram of CDs) (**b**), and CDs/FeCo-ONSs (**c**).

The morphologies of FeCo-ONSs, CDs, and CDs/FeCo-ONSs were characterized by HRTEM. FeCo-ONSs (Figure 1a) exhibit a well-defined sheet-like morphology, corresponding to a typical two-dimensional lamellar structure. The hierarchical architecture with a high aspect ratio endows the composite with an enlarged specific surface area and facilitates the construction of efficient electron transport pathways. In Figure 1b, the CDs exhibit a relatively uniform spherical nanostructure with a diameter of 3.8 nm. Figure 1c clearly reveals that the sheet-like structure of FeCo-ONSs and the quasi-spherical CDs yield distinct composite morphological characteristics, and the spherical CDs are uniformly distributed on the FeCo-ONSs’ surface, verifying the effective construction of the composite.

To further characterize the vertical dimension and surface morphology of the nanomaterials, AFM was employed to investigate FeCo-ONSs and CDs/FeCo-ONSs. As shown in Figure S1, FeCo-ONSs exhibit a typical two-dimensional nanosheet morphology with a smooth surface and no obvious attached particles. The height profile curve reveals that the sheet thickness is approximately 3.2–3.5 nm, demonstrating the successful synthesis of ultrathin two-dimensional nanosheets. Such ultrathin features provide abundant surface-active sites and electron transport channels for subsequent composite modification. For CDs/FeCo-ONSs, numerous uniformly distributed protrusive particles appear on the originally smooth nanosheet surface, corresponding to the loaded CDs. The height profile curve shows that the sheet thickness increases to 6.2–6.5 nm after composition, which directly confirms that CDs have been successfully anchored on the surface of FeCo-ONSs.

To further evaluate the pore structure of the materials, nitrogen adsorption–desorption isotherms were measured. As shown in Figure S2, CDs/FeCo-ONSs exhibit a typical Type IV isotherm, indicating the presence of a mesoporous structure. The BET-specific surface area of CDs/FeCo-ONSs is 85.6 m^2^/g, which is distinctly higher than that of pure FeCo-ONSs 72.2 m^2^/g. The increase in specific surface area is mainly attributed to the intercalation of CDs into the nanosheet interlayers, which effectively inhibits the stacking and aggregation of FeCo-ONSs. This highly open structure provides abundant contact sites for H_2_O_2_ and TMB and accelerates the mass transfer process, thereby endowing the composite with excellent enzyme-like catalytic performance.

**Figure 2:**
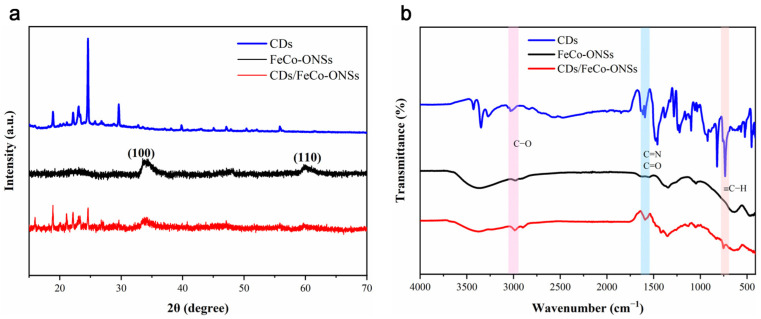
XRD patterns of CDs, FeCo-ONSs, and CDs/FeCo-ONSs (**a**), and FT-IR spectra of CDs, FeCo-ONSs, and CDs/FeCo-ONSs (**b**).

The crystal structures of CDs, FeCo-ONSs, and CDs/FeCo-ONSs were investigated by X-ray diffraction (XRD), as shown in Figure 2a. The XRD pattern of CDs displays distinct diffraction peaks in the 20° to 30° 2θ range, which reflects the presence of a short-range ordered crystalline carbon framework within their structure and thus confirms a certain degree of crystallinity for CDs, which is consistent with the results observed in the HRTEM images. The XRD pattern of FeCo-ONSs exhibits two weak diffraction peaks at 33.8° and 59.8°, which correspond to the (100) and (110) crystal planes, respectively (JCPDS No. 14-0191). This result indicates that FeCo-ONSs possess relatively low crystallinity and typical ultra-thin two-dimensional (2D) characteristics. The XRD pattern of CDs/FeCo-ONSs exhibits a combined diffraction profile, retaining characteristic peak positions of CDs and FeCo-ONSs: there is a signal response at the angular positions corresponding to the characteristic peaks of CDs, while the diffraction peaks of FeCo-ONSs at their respective angles are also manifested. However, the intensity and morphology of each peak changed significantly, and some characteristic peaks even disappeared completely. The disappearance of the diffraction peak of FeCo-ONSs at 59.8° indicates that the nanosheets are effectively encapsulated by carbon dots, resulting in the shielding of part of the diffraction planes. These modifications indicate mutual interaction between the two components upon composite formation, which alters their original crystalline environments. Such structural evolution provides direct evidence for the successful construction of the CDs/FeCo-ONSs’ heterostructure.

**Figure 3:**
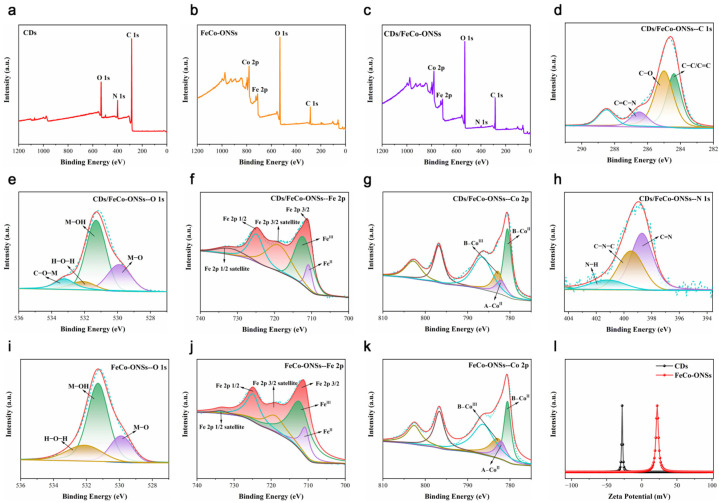
XPS patterns of CDs (**a**), FeCo-ONSs (**b**), and CDs/FeCo-ONSs (**c**). High-resolution XPS spectra of C 1s (**d**), O 1s (**e**), Fe 2p (**f**), Co 2p (**g**), and N 1s (**h**) in CDs/FeCo-ONSs. High-resolution XPS spectra of O 1s (**i**), Fe 2p (**j**), and Co 2p (**k**) in FeCo-ONSs. Zeta potential of CDs and FeCo-ONSs (**l**).

Figure 2b illustrates the surface functional group analysis of CDs, FeCo-ONSs, and CDs/FeCo-ONSs via FT-IR spectroscopy. The spectrum of CDs displays that the absorption peak around 3000 cm^−1^ is assigned to the stretching vibration of C–O bonds; additionally, characteristic vibration peaks of C=N and C=O bonds appear at approximately 1500 cm^−1^. These features indicate that oxygen- and nitrogen-containing functional groups were abundant on the surface of CDs. Regarding FeCo-ONSs, the broad absorption peak at 3379 cm^−1^ is attributed to the stretching vibration of O-H bonds on the FeCo-ONSs’ surface. The FT-IR of CDs/FeCo-ONSs exhibits partial characteristic absorption peaks of both CDs and FeCo-ONSs, yet significant variations in peak intensity and peak shape are observed. Specifically, the attenuation and redshift of the C–O stretching vibration, whose peak intensity is lower than that of pristine CDs, indicates the generation of C–O–M coordination bonds between the C–O groups of the CDs and the metal (M) sites of the FeCo-ONSs, accompanied by a corresponding decrease in bond energy (Figure 3e). Additionally, in the range of C=N and C=O bond characteristic peaks, the absorption peaks become broadened with reduced intensity, and the nitrogen- and oxygen-containing functional groups of CDs participate in interfacial bonding, leading to a decrease in the degree of freedom of these functional moieties. These spectral changes collectively provide compelling evidence for the successful fabrication of the CD/FeCo-ONS nanocomposite.

Specifically, the reduced intensity of the C–O stretching band compared to pristine CDs suggests alterations in the chemical environment of these functional groups following composite formation. Moreover, the broadening and diminished intensity of the characteristic stretching peaks of C=N and C=O provide additional spectroscopic evidence for the successful fabrication of the CDs/FeCo-ONSs hybrid material. These observations collectively support the proposed structural configuration of the composite system.

Figure 3a–c present the X-ray photoelectron spectroscopy (XPS) survey spectra of CDs, FeCo-ONSs, and CDs/FeCo-ONSs, respectively. CDs contain C, N, and O elements, FeCo-ONSs consist of Co, Fe, and O elements, while the CD/FeCo-ONS composite additionally exhibits the presence of N from carbon dots. Figure 3g shows three characteristic binding energy peaks of Co 2p3/2. Here, the 781.1 eV and 782.9 eV peaks are attributed to Co(II) and Co(III) in octahedral coordination, while the peak at 782.1 eV is assigned to Co(II) in tetrahedral coordination. The Fe 2p spectrum (Figure 3f) presents peaks at 710.8 eV (Fe 2p3/2 of Fe(II)), 712.2 eV (Fe 2p3/2 of Fe(III)), and 724.9 eV (Fe 2p1/2). Following the loading of CDs, the binding energy of the CD/FeCo-ONS nanocomposite exhibits a discernible shift. After loading CDs, the molar ratios of Fe^3+^/Fe^2+^ and Co^3+^/Co^2+^ increased significantly, rising from 0.4 to 0.8 and from 3.5 to 5.1, respectively. This variation confirms that efficient electron transfer occurs between CDs and FeCo-ONSs during the ultrasonic composite process, which induces the formation of a higher proportion of high-valence metal ions. High-valence metal ions are generally regarded as the main active centers for catalyzing the decomposition of H_2_O_2_ to generate ·OH radicals. These findings collectively demonstrate that CDs and FeCo-ONSs achieve interfacial coupling via chemical bonding during the ultrasonic treatment process. The C 1s spectrum (Figure 3d) deconvolutes into three peaks at 284.4 eV, 285.0 eV, and 286.5 eV, attributable to C–C/C=C, C–O, and C=C–N groups. Similarly, the N 1s spectrum (Figure 3h) exhibits three characteristic peaks at 398.7 eV, 399.5 eV, and 401.2 eV, assigned to C=N, C–N–C, and N–H moieties, respectively [17,18]. This phenomenon can be ascribed to the introduction of CDs featuring abundant surface-bounded functional groups. Figure 3e exhibits four peaks at 529.9 eV, 531.3 eV, 532.1 eV, and 533.3 eV, assigned to M–O, M–OH, H–O–H, and C–O–M moieties [29]. In comparison with spectrum Figure 3i, there is an enhancement in the characteristic peak intensity of C–O–M as well as a reinforcement in the bond strength of M–O. The obtained test results corroborate that CDs are successfully immobilized onto FeCo-ONSs via chemical bonding, which is driven by the strong electrostatic attractions between cationic FeCo-ONSs and anionic CDs (Figure 3l). CDs accelerate the electron exchange between Fe/Co active centers and substrates (TMB/H_2_O_2_). The binding energy shifts observed in XPS spectra directly reflect such strong electronic interaction, which further reduces the activation energy of the catalytic reaction.

To further investigate the interfacial charge dynamics between CDs and FeCo-ONSs, steady-state photoluminescence (PL) and time-resolved photoluminescence (TRPL) analyses were performed. As shown in Figure S3, pure CDs exhibit an intense emission peak at 375 nm. However, upon the introduction of FeCo-ONSs, the fluorescence intensity of CDs is significantly quenched. This quenching phenomenon is generally attributed to the efficient charge transfer process from the excited state of CDs to the surface-active sites of FeCo-ONSs. In addition, the TRPL spectra demonstrate that the fluorescence decay curve of CDs fits well with a double-exponential function. After compositing with FeCo-ONSs, the average fluorescence lifetime of CDs decreases from 1.8 ns to 1.4 ns. The reduction in lifetime directly confirms the existence of interfacial charge transfer, which not only suppresses the radiative recombination of carriers but also promotes the efficient transfer of electrons at the interface of the composite, thereby lowering the energy barrier for the decomposition of H_2_O_2_ to generate ·OH.

### 3.2. POD-like Activity of CDs/FeCo-ONSs

The fabricated CDs/FeCo-ONSs nanocomposite exhibits favorable POD-like activity. Here, TMB was utilized as the catalytic oxidation substrate with H_2_O_2_. Figure S4 systematically investigates the influence of key reaction parameters (pH, reaction time, and reaction temperature) on the catalytic activity of CDs/FeCo-ONSs. The results demonstrate that optimal enzymatic activity was achieved at a pH of 3.5, a reaction time of 35 min, and a reaction temperature of 50 °C.

**Figure 4:**
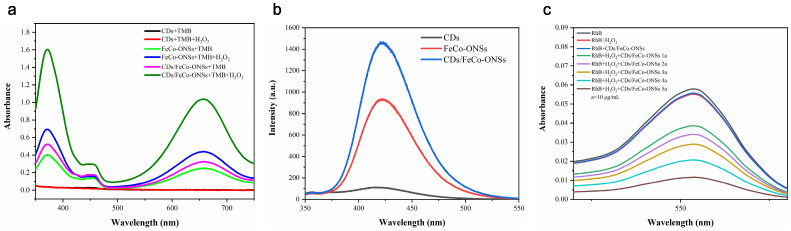
UV-Vis absorption spectra of the mixture of CDs, FeCo-ONSs, CDs/FeCo-ONSs, H_2_O_2_, and TMB (**a**). Fluorescence spectra of HTA products in reactions verified by different substances (**b**). UV-Vis absorption spectra of RhB catalyzed by CDs/FeCo-ONSs with different contents (10, 20, 30, 40, and 50 μg/mL) in the presence of H_2_O_2_ (**c**).

Figure 4a displays the UV-Vis absorption spectra of various reaction systems under optimized reaction conditions. Upon comparative analysis, the CDs/FeCo-ONSs composite exhibits significantly higher absorbance than its individual components and other reference materials. This significant enhancement in absorption intensity confirms the excellent POD-like activity of the nanocomposite, suggesting a synergistic effect among CDs and FeCo-ONSs.

The POD-like catalytic pathway can be categorized into electron transfer processes and hydroxyl radical (·OH) generation. To investigate the active intermediates in the catalysis system, fluorescence and colorimetric probes were utilized. First, TA served as a fluorescent probe for ·OH, whereby it reacts with hydroxyl radicals to form highly fluorescent 2-hydroxyterephthalic acid, allowing for the quantitative monitoring of ·OH generation. Figure 4b reveals the presence of a prominent characteristic peak at 426 nm in the CD/FeCo-ONS system, which verifies the production of ·OH. Notably, the CD/FeCo-ONS nanocomposite showed a stronger fluorescence signal than the individual components, reflecting that synergistic effects between CDs and FeCo-ONSs boost ·OH generation efficiency and drive the superior POD-like activity of the CD/FeCo-ONS nanocomposite.

For further verification of the POD-catalyzed reaction mechanism of CDs/FeCo-ONSs, a RhB decolorization assay was conducted to track ·OH formation. As illustrated in Figure 4c, the absorbance of RhB at 554 nm decreased significantly in the H_2_O_2_-containing system compared to the control experiment, indicating the degradation of RhB by generated ·OH. Moreover, as the concentration of CDs/FeCo-ONSs increased, a more pronounced absorbance decrease at 554 nm was evident, confirming the presence of ·OH during the catalytic process. Additionally, it can be observed that the catalytic activity is dependent on the concentration of CDs/FeCo-ONSs.

### 3.3. Kinetic Investigation of POD-like Activity

**Figure 5:**
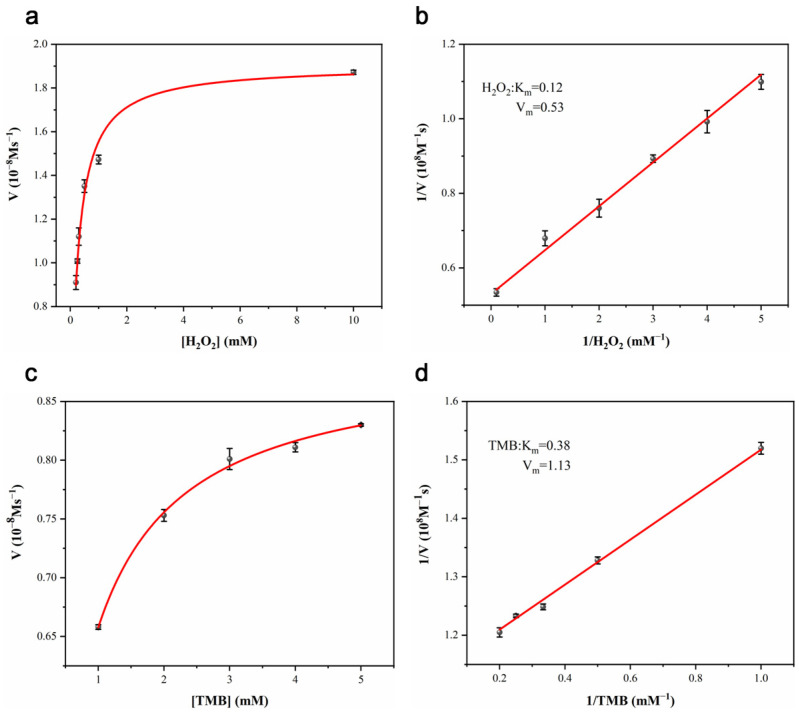
Steady-state kinetics analysis of CDs/FeCo-ONSs. Plot of initial reaction velocity versus H_2_O_2_ concentration (**a**). Lineweaver-Burk double-reciprocal plot derived from the H_2_O_2_ titration data (**b**). Plot of initial reaction velocity versus TMB concentration (**c**). Lineweaver–Burk double–reciprocal plot derived from the TMB titration data (**d**).

To investigate the POD-like activity of CDs/FeCo-ONSs, steady-state kinetics experiments were explored under the aforementioned optimized conditions by varying the concentrations of H_2_O_2_ and TMB. As illustrated in Figure 5a,c, the kinetic curves for both H_2_O_2_ and TMB are well-fitted to the classic Michaelis–Menten model.

The corresponding Lineweaver–Burk plots derived from the kinetic data are presented in Figure 5b,d, allowing for the calculation of the Michaelis–Menten constant (K_m_) and maximum reaction rate (V_m_). Specifically, the K_m_ values of CDs/FeCo-ONSs for H_2_O_2_ and TMB were determined to be 0.12 mM and 0.38 mM, respectively. These results indicate that CDs/FeCo-ONSs exhibits an enhanced affinity toward both H_2_O_2_ and TMB compared to horseradish peroxidase (HRP), whose K_m_ values for the two substrates are 3.7 mM and 0.434 mM, respectively.

A smaller K_m_ indicates a stronger affinity between the catalyst and its substrates. The kinetic parameters of CDs/FeCo-ONSs were compared with those of reported nanozymes in the literature, as summarized in Table 1, highlighting the superior catalytic activity of the CD/FeCo-ONS composite.

### 3.4. Colorimetric Sensing of H_2_O_2_ and AA

**Figure 6:**
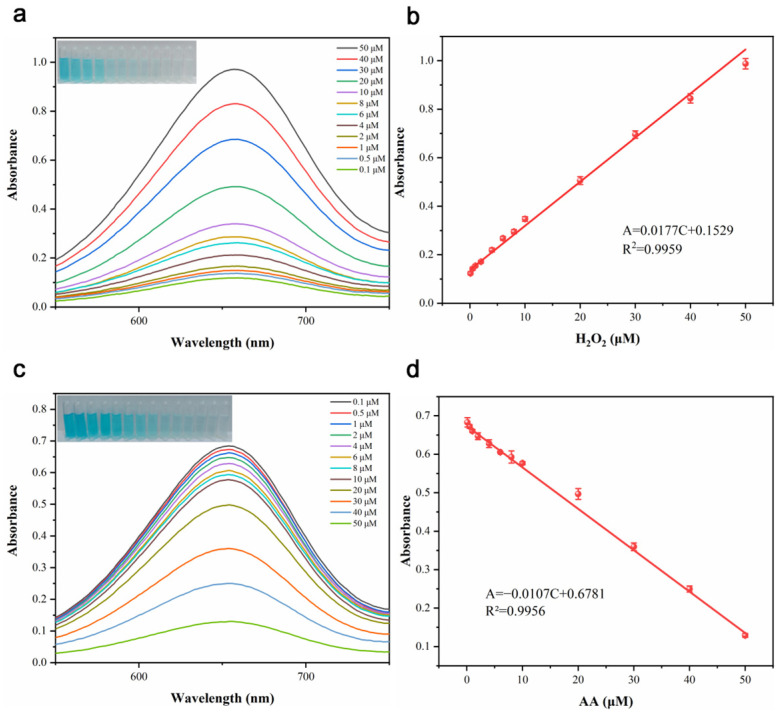
UV-Vis spectra changes of CDs/FeCo-ONSs with different H_2_O_2_ concentrations, with the inset showing the corresponding color gradation (**a**), linear calibration curve for H_2_O_2_ in the range of 0.1–50 μM (**b**), UV-Vis spectra changes for AA detection (0.1–50 μM) with photographic insets (**c**), and linear calibration plot for AA over the same concentration range (**d**).

Based on the POD-like catalytic activity of CDs/FeCo-ONSs, TMB undergoes oxidation to its blue oxidized product in the coexistence of H_2_O_2_ and the nanocomposite. This oxidation process is concentration dependent and allows for the colorimetric detection of H_2_O_2_ based on the corresponding color change. As depicted in Figure 6a, a gradual color fade from blue to colorless was observed for the reaction mixture with a decrease in H_2_O_2_ concentration. Meanwhile, Figure 6b reveals a good linear relationship between absorbance and H_2_O_2_ concentration (0.1–50 µM), with the linear regression equation being A = 0.0177C + 0.1529 (R^2^ = 0.9959, where A represents the absorbance and C is the H_2_O_2_ concentration, and the standard errors of the slope and intercept are 3.45 × 10^−4^ and 74.6 × 10^−4^, respectively), with a LOD of 0.036 µM determined for H_2_O_2_.

Due to its reducing properties, AA can reduce the blue oxidized form of TMB back to its colorless form. Consequently, a colorimetric method for AA detection can be established based on this reduction reaction. Figure 6c,d show that TMB absorbance at 652 nm declined progressively with the increasing AA concentration. A significant linear relationship (R^2^ = 0.9956) is found among the change in TMB absorbance and AA concentration in the 0.01–50 µM range, following the regression equation A = −0.0107C + 0.6781 (where A is TMB absorbance at 652 nm and C is AA concentration, and the standard errors of the slope and intercept are 1.56 × 10^−4^ and 26.7 × 10^−4^, respectively). The LOD for AA is calculated as 0.018 µM, which outperforms the reported LODs for AA detection in other literature, as compiled in Table 2. The LOQ for AA is calculated as 0.06 µM. It was found through comparison that the synthesis time of CDs/FeCo-ONSs is relatively long. However, the synthesis process involves hydrothermal reaction, dialysis purification, and drying, and this sophisticated preparation process endows the material with excellent colorimetric sensing performance. In particular, its detection limit for AA reaches 0.018 µM, which is lower than that of many existing colorimetric methods. This extremely high sensitivity offsets the cost in synthesis time, enabling it to be competent for the detection of real samples with extremely high requirements for trace analysis. In addition, the interface stability achieved through chemical bonding ensures that the material has excellent anti-interference ability in complex food matrices, reflecting its unique application value as a high-performance nanozyme.

To evaluate the precision of the CDs/FeCo-ONSs colorimetric method, intra-day and inter-day repeatability were investigated. Four concentrations of ascorbic acid (5, 10, 20, and 30 µM) were determined via five parallel measurements with three replicates for each group. The intra-day RSDs were calculated to be 0.94–2.2% (Table S1). Furthermore, 30 µM of AA was measured over five consecutive days, yielding inter-day RSDs of 1.0–1.7% (Table S2). These results demonstrate that the sensing platform possesses excellent reproducibility and reliability for the quantitative analysis of AA.

To investigate the robustness of the analytical method, slight intentional variations of experimental conditions were evaluated in the detection system containing 20 μM of AA. For the detection of 20 μM of AA, the pH was varied from 3.3 to 3.7 around the optimized value of 3.5, and the incubation temperature was varied from 48 to 52 °C around the optimized value of 50 °C. The experimental results showed that the RSDs of the response signals were in the range of 1.1–2.8% (Tables S3 and S4). This confirms that the CDs/FeCo-ONSs sensing platform possesses favorable robustness and can tolerate minor fluctuations that may occur in routine laboratory operations.

The operational stability of the composite was further evaluated by recycling experiments. As shown in Figure S5, the CD/FeCo-ONS composite retained its peroxidase-like activity after six repeated cycles, suggesting that no obvious activity loss occurred during repeated analytical use. These results indicate that the interfacial coordination and surface protection effect of CDs contribute to the stability of the Fe-containing composite under the present sensing conditions.

To evaluate the selectivity of the CD/FeCo-ONS-based sensing system, the impact of potential interfering species on the colorimetric detection of AA was investigated. A series of common metal ions, inorganic anions, and other biomolecules were selected as interfering analytes, including K^+^, Ca^2+^, Mg^2+^ Na^+^, I^−^, sucrose, fructose, glucose, glycine, and L-tryptophan. As illustrated in Figure S6, even when the concentration of the introduced interfering substances was 100-fold higher than that of AA, the catalytic reaction remained unaffected. These results demonstrate that the CD/FeCo-ONS-based detection system exhibits excellent selectivity and specificity for AA detection, making it suitable for complex analytical applications.

To ascertain the practical feasibility of this colorimetric detection method, three beverages (Nongfu Spring C100 juice, Master Kong Daily C Peach juice, and Minute Maid Orange juice) and three fresh fruits (litchi, kiwifruit, and orange) were selected as real samples for detection. The results showed that the measured concentrations of the diluted beverage and fruit juice samples were consistent with those of the actual samples. To verify the accuracy of the colorimetric method, the experimentally determined AA concentrations were compared with the nominal values marked on the beverage packages. The original AA concentrations of Nongfu Spring C100 Juice, Master Kong Daily C Peach Juice, and Minute Maid Orange Juice were 1300 μM, 1100 μM, and 400 μM, respectively. After conversion according to the dilution factor, the measured AA contents were in good agreement with the nutritional information stated on the product labels. Although the intrinsic AA concentration in real samples falls within the linear detection range of this method, standard spike recovery experiments were further carried out to evaluate the matrix effect and verify the accuracy of the sensing platform. Different concentrations of standard AA solutions were spiked into samples with known endogenous AA contents, and the corresponding recoveries were calculated. The results effectively demonstrate the reliability of this method against the interference of complex food components. As presented in Table 3, the spike recovery rates of AA ranged from 95.0% to 102%, with corresponding RSDs all within 2.8%. These findings verify that this developed colorimetric detection method exhibits excellent accuracy and reliability for the detection of AA in complex real samples.

To further validate the accuracy of the proposed method, we performed an additional comparison study using the direct iodometry method for AA determination. As listed in Table S5, the measured AA concentrations in Nongfu Spring C100 Juice, Master Kong Daily C Peach Juice, and Minute Maid Orange Juice were 9.92 μM, 9.99 μM, and 10.07 μM, respectively, with the RSDs of three parallel measurements all lower than 2%. The results obtained by iodometry were in good consistency with the AA concentrations determined via the spiked recovery method of this work, demonstrating that the established detection method possesses favorable and reliable accuracy.

The sensing mechanism of the CDs/FeCo-ONSs colorimetric platform can be explained by the peroxidase-like catalytic activity of the composite and the reducing ability of AA. In the presence of H_2_O_2_, the Fe/Co redox-active centers in CDs/FeCo-ONSs catalyze the decomposition of H_2_O_2_ to generate reactive oxygen species, particularly ·OH, which oxidize colorless TMB into blue oxTMB with a characteristic absorbance at 652 nm. The CDs coupled on the surface of FeCo-ONSs through interfacial C–O–M bonding improve the dispersion of the nanosheets and may promote interfacial electron transfer, thereby enhancing the catalytic oxidation of TMB. For AA detection, AA reduces blue oxTMB back to colorless TMB, leading to a concentration-dependent decrease in absorbance at 652 nm. Radical scavenging experiments confirmed the dominant role of ·OH in TMB oxidation, while kinetic analysis demonstrated the high affinity of CDs/FeCo-ONSs toward H_2_O_2_. These results collectively support the proposed H_2_O_2_/AA colorimetric sensing mechanism.

## 4. Conclusions

In this work, we successfully synthesized yellow-emitting nitrogen-doped CDs through a one-pot hydrothermal preparation strategy and fabricated the CDs/FeCo-ONSs nanocomposite through an ultrasound-assisted impregnation approach. The as-fabricated CD/FeCo-ONS composite exhibited enhanced peroxidase-like activity, which efficiently catalyzed the oxidation of the chromogenic substrate TMB to its blue oxidized form in the presence of H_2_O_2_. Based on this catalytic mechanism, a novel colorimetric sensing platform was established with a wide linear range and low limit of detection. The strategy proposed in this study exhibits excellent selectivity and accuracy for the detection of AA, as validated through successful analysis of real beverage and fruit samples.

Although the synthesis process of CDs/FeCo-ONSs involves hydrothermal reaction, dialysis purification, and prolonged drying, this elaborate preparation process endows the material with excellent colorimetric sensing performance. In particular, its LOD for AA reaches 0.018 µM, which is much lower than that of many existing colorimetric methods. This extremely high sensitivity offsets the cost of the long synthesis time, enabling the material to be competent for the detection of real samples with extremely high requirements for trace analysis. In addition, the interfacial stability achieved through chemical bonding ensures that the material possesses excellent anti-interference ability in complex food matrices, reflecting its unique application value as a high-performance nanozyme. This work provides a convenient and reliable new method for the detection of AA in food and shows great application potential in the field of food safety monitoring.

## Figures and Tables

**Table 1:** Kinetic behavior comparisons of various nanozyme catalysts.

Catalyst	K_m_ (mM)		V_m_ (10^−8^ M·s^−1^)		Reference
	TMB	H_2_O_2_	TMB	H_2_O_2_	
HRP	0.434	3.7	10	8.71	[39]
Pd-CDs	0.74	10.12	4.6	0.72	[40]
Zr-MOFs@Pt	0.34	1.52	9.98	7.09	[41]
Fe-BTC	0.45	0.38	2.38	1.29	[42]
CoFe_2_O_4_	0.37	8.89	2.09	1.93	[43]
CDs/FeCo-ONSs	0.38	0.12	1.13	0.53	This work

**Table 2:** Comparison of colorimetric sensing assays: CDs/FeCo-ONSs vs. previously reported methods for AA detection.

Probe	Synthetic Route	Time (h)	Linear Range (μM)	LOD	Reference
C/NiFe_2_O_4_	Hydrothermal	-	1–25	0.26	[44]
Co-CQD	Solvothermal	>46.5	10–400	0.27	[45]
Fe_3_O_4_@Au/MOF	In situ growth	66	1–100	0.098	[46]
N,Fe-CDs	Hydrothermal	46	5–50	2.05	[47]
Co_3_O_4_/CGM	Pyrolysis	>48	30–140	0.19	[48]
Pt/CeO_2_	Chemical reduction	41	0.5–30	0.08	[49]
CDs/FeCo-ONSs	Ultrasonic-assisted integration	55	0.1–50	0.018	This work

**Table 3:** The results and spike recovery rates of AA determination in beverages and fresh fruits.

Samples	Dilution	Added AA Amount (µM)	Colorimetric (µM)	Recovery (%)	RSD (*n* = 3, %)
Nongfu Spring C100	130	0	9.93	—	—
		10	20.0	101%	1.1%
		15	24.9	99.8%	1.8%
Master Kong Daily C Peach Juice	110	0	9.02	—	—
		10	19.0	99.8%	1.2%
		15	24.5	103%	2.8%
Minute Maid Orange	40	0	10.8	—	—
		10	21.1	103%	1.2%
		15	25.4	96.9%	1.6%
Litchi	200	0	19.8	—	—
		20	39.0	96.0%	1.4%
		30	49.1	97.8%	1.1%
Kiwifruit	130	0	18.6	—	—
		20	38.1	97.4%	2.3%
		30	49.1	102%	1.9%
Orange	90	0	19.9	—	—
		20	38.9	95.0%	2.4%
		30	49.2	97.5%	1.2%

## Data Availability

The original contributions presented in this study are included in the article/Supplementary Material. Further inquiries can be directed to the corresponding author.

## Supplementary Materials

The following supporting information can be downloaded at: https://www.mdpi.com/article/10.3390/nano16100634/s1. Figure S1. AFM image (a) and height profile (b) of FeCo-ONSs, AFM image (c) and height profile (d) of CDs/FeCo-ONSs. Figure S2. N_2_ adsorption-desorption isotherms of FeCo-ONSs (a) and CDs/FeCo-ONSs (b). Figure S3. Steady-state fluorescence spectra (a) and transient fluorescence spectra (b) of pure CDs and CDs/FeCo-ONSs. Figure S4. Optimization of the catalytic conditions for the CDs/FeCo-ONSs nanozyme: pH (a), incubation time (b), and reaction temperature (c) on the oxidation of TMB under standard conditions. Figure S5. The cyclic reusability stability of CDs/FeCo-ONSs was investigated. Using TMB as the substrate in the presence of H_2_O_2_, the changes in UV–Vis absorption spectra were measured over seven consecutive recycling runs. Figure S6. UV-Vis absorbance for the the selective detection of AA by CDs/FeCo-ONSs. Table S1. Intra-day repeatability of AA detection by CDs/FeCo-ONSs colorimetric method. Table S2. Inter-day repeatability of AA detection by CDs/FeCo-ONSs colorimetric method (30 µM AA). Table S3. Robustness evaluation of the CDs/FeCo-ONSs colorimetric method for detecting 20 μM AA under different pH conditions. Table S4. Robustness evaluation of the CDs/FeCo-ONSs colorimetric method for detecting 20 µM AA under different incubation temperatures. Table S5. Determination of AA by direct iodometry.
