# Supplementary material for: CDs/FeCo-ONSs Composite with Peroxidase-like Activity for Ascorbic Acid Detection
Source: Nanomaterials (Basel). 2026 May 20;16(10):634. doi: 10.3390/nano16100634 (PMC13209517; doi:10.3390/nano16100634)
Supplement: Supplementary file 1 [file nanomaterials-16-00634-s001.zip › nanomaterials-4288299-supplementary.pdf]

Supporting Information for

CDs/FeCo-ONSs Composite with Peroxidase-like Activity for Ascorbic Acid Detection

Liu Xue, Wei Yuanhang, Wang Wenjing*

College of Chemistry and Chemical Engineering, Qingdao University, Qingdao 266071, China

* *Correspondence: wangwenjing@qdu.edu.cn*

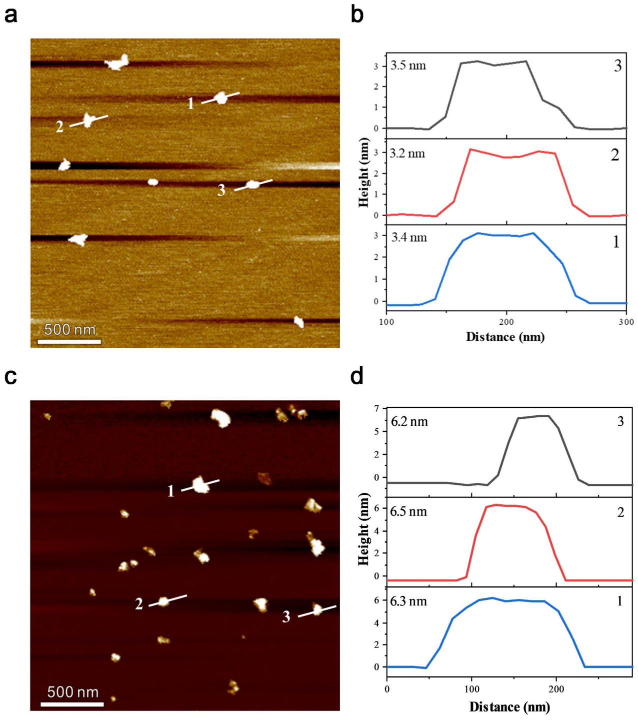

Figure S1. AFM image (a) and height profile (b) of FeCo-ONSs, AFM image (c) and height profile (d) of CDs/FeCo-ONSs.

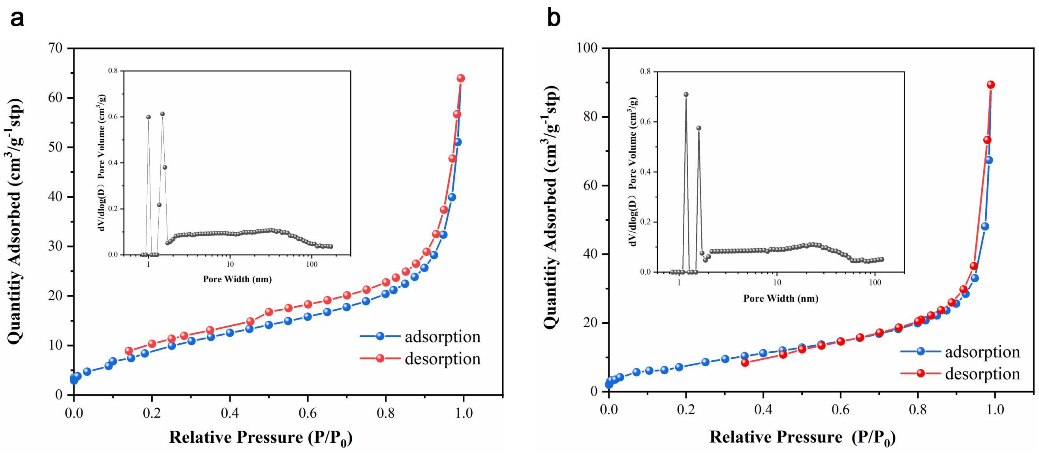

Figure S2. N_2 adsorption-desorption isotherms of FeCo-ONSs (a) and CDs/FeCo-ONSs (b).

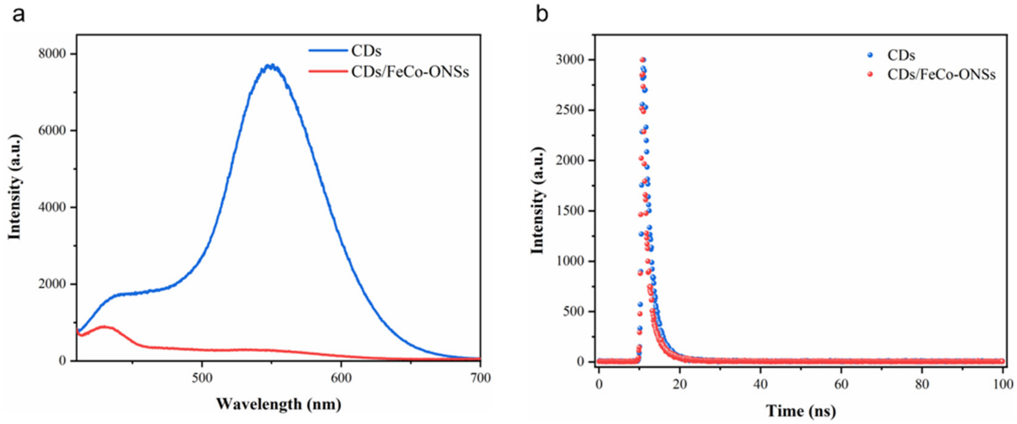

Figure S3. Steady-state fluorescence spectra (a) and transient fluorescence spectra (b) of pure CDs and CDs/FeCo-ONSs.

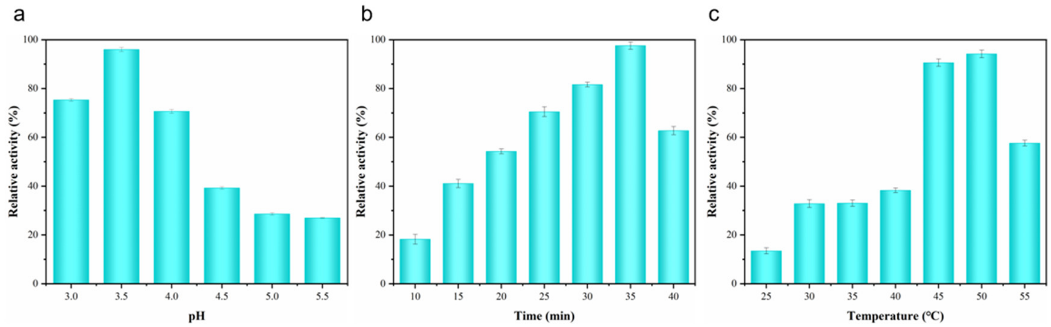

Figure S4. Optimization of the catalytic conditions for the CDs/FeCo-ONSs nanozyme: pH (a), incubation time (b), and reaction temperature (c) on the oxidation of TMB under standard conditions.

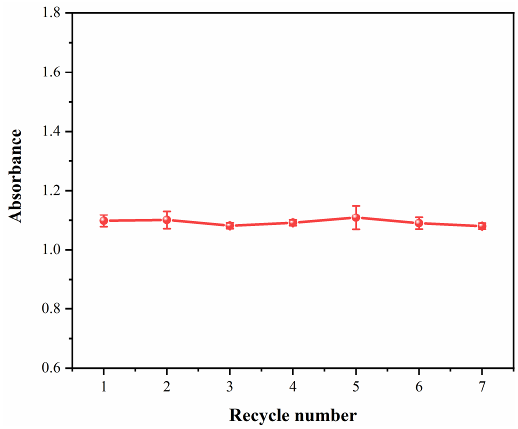

Figure S5. The cyclic reusability stability of CDs/FeCo-ONSs was investigated. Using TMB as the substrate in the presence of H_2O_2, the changes in UV–Vis absorption spectra were measured over seven consecutive recycling runs.

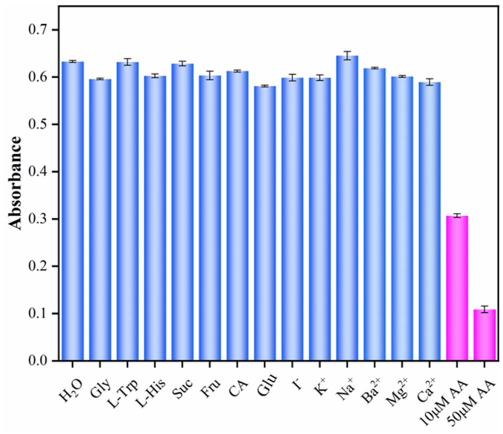

Figure S6. UV-Vis absorbance for the the selective detection of AA by CDs/FeCo-ONSs.

Table S1. Intra-day repeatability of AA detection by CDs/FeCo-ONSs colorimetric method.

**AA Concentration (μM)**	**Time**					**RSD (%)**
	**1**	**2**	**3**	**4**	**5**	
**4**	0.6212	0.6198	0.6311	0.5951	0.6132	2.2
**10**	0.5784	0.5812	0.5698	0.5798	0.5703	0.94
**20**	0.4874	0.4956	0.4932	0.4802	0.4932	1.3
**30**	0.3674	0.3588	0.3654	0.3621	0.3599	1.0

Table S2. Inter-day repeatability of AA detection by CDs/FeCo-ONSs colorimetric method (30 μM AA).

**Group**	**Day**					**RSD (%)**
	**1**	**2**	**3**	**4**	**5**	
**1**	0.3604	0.3668	0.3589	0.3643	0.3673	1.0
**2**	0.3568	0.3698	0.3678	0.3574	0.3602	1.7
**3**	0.3678	0.3601	0.3636	0.3701	0.3652	1.1

Table S3. Robustness evaluation of the CDs/FeCo-ONSs colorimetric method for detecting 20 μM AA under different pH conditions.

Group / pH	3.3	3.5	3.7	RSD (%)
**1**	0.4984	0.5101	0.5023	1.2
**2**	0.4901	0.5175	0.4988	2.8
**3**	0.4943	0.5054	0.5010	1.1

Table S4. Robustness evaluation of the CDs/FeCo-ONSs colorimetric method for detecting 20 μM AA under different incubation temperatures.

Group / T/°C	48	50	52	RSD (%)
**1**	0.4889	0.5008	0.4930	1.2
**2**	0.4960	0.5103	0.4983	1.5
**3**	0.4991	0.5089	0.4967	1.2

Table S5. Determination of AA by direct iodometry.

	Nongfu Spring C100 Juice	Master Kong Daily C Peach Juice	Minute Maid Orange Juice
Average volume of consumed I_2 (mL)	9.92	9.99	10.07
RSD (n = 3, %)	1.19	1.17	1.41
Measured AA concentration (μM)	9.92	9.99	10.07
